# Exploring the applicability of a lesion segmentation method on [^18^F]fluorothymidine PET/CT images in diffuse large B-cell lymphoma

**DOI:** 10.1186/s41824-023-00184-3

**Published:** 2023-12-25

**Authors:** Germán Pitarch, Yamila Rotstein Habarnau, Roxana Chirico, Brenda Konowalik, Amalia Pérez, Alejandro Valda, María Bastianello

**Affiliations:** 1 Sección de Imágenes Moleculares y Terapia Metabólica, Hospital Universitario CEMIC, Ciudad Autónoma de Buenos Aires, Argentina; 2 Centro Universitario de Imágenes Médicas, Escuela de Ciencia y Tecnología, Universidad Nacional de San Martín, Buenos Aires, Argentina

**Keywords:** Non-Hodgkin’s lymphoma, PET/CT, Fluorine-18 fluorothymidine, Segmentation, Tumour proliferation volume

## Abstract

**Background and purpose:**

The determination of the total metabolic tumour volume based on [^18^F]fluorothymidine ([^18^F]FLT) PET/CT images in diffuse large B-cell lymphoma has a potential clinical value for detecting early relapse in this type of heterogeneous lymphoproliferative tumours. Tumour segmentation is a key step in this process. For this purpose, our objective was to determine a segmentation threshold of [^18^F]FLT PET/CT images, based on a reference tissue uptake, on a cohort of patients with diffuse large B-cell lymphoma (DLBCL) that have been scanned at different stages of the treatment.

**Methods:**

We enrolled 23 adult patients with DLBCL confirmed in II-IV stages without nervous system compromise. All patients were scanned using [^18^F]FLT PET/CT at the time of diagnosis (baseline PET), interim PET (iPET), and at the end of treatment (fPET). The administered activity was 1.8–2.6 MBq/kg body weight, performed 60–70 min after injection and without use of contrast-enhanced CT. First, we assessed the [^18^F]FLT uptake stability in liver and bone marrow along the patient follow-up. For the lesion segmentation, three threshold values were assessed.

**Results:**

Both, liver, and bone marrow can be indistinctly taken as reference tissue. The SUV threshold for a voxel to be considered as belonging to a lesion is expressed in terms of a percentage relative to the patient’s uptake in the reference tissue. Found thresholds were: for liver, 62%, 33%, 27%; and for bone marrow, 35%, 21% and 22%, for baseline, iPET and fPET stages, respectively. The relative threshold throughout the treatment has a decreasing tendency along the stages.

**Conclusion:**

Based on the results obtained with [^18^F]FLT PET/CT during staging and follow-up in patients with DLBCL, reference values were obtained for each stage referring to liver and bone marrow uptake that could be used in clinical practice oncology.

## Background

Diffuse large B-cell lymphoma (DLBCL) is the most common subtype of non-Hodgkin’s lymphoma. Although the cure rate of DLBCL has improved with R-CHOP immunotherapy treatments, over 30–40% of the patients relapse or do not respond to this treatment (Mikhaeel et al. 2022; Mengüç et al. 2021). Fluorine-18 fluorodeoxyglucose ([^18^F]FDG) PET/CT imaging is nowadays the standard procedure for staging and restaging of DLBCL. This modality, however, has a high false positive rate, related to residual inflammatory processes. Due to this lack of specificity, [^18^F]FDG PET/CT may not be the best method for monitoring the response to the treatment (Spaepen et al. 2003).

Fluorine-18 fluorothymidine ([^18^F]FLT) has emerged as a marker of cellular proliferation (McKinley et al. 2013) whose uptake is less affected by underlying inflammatory processes after therapy and might therefore represent a more tumour-specific marker (Buck et al. 2006). Recent trials have shown that [^18^F]FLT is a superior predictor compared to [^18^F]FDG (Mengüç et al. 2021; Minamimoto et al. 2016).

The total metabolic tumour volume (MTV) has been proposed as a promising biomarker of outcome in DLBCL (Sasanelli et al. 2014; Song et al. 2012) as well as for other types of lymphoma. However, tumour segmentation in lymphoma studies is not a simple process in the context of a usually complex scenario involving multiple lesions of diverse sizes and shapes in addition to a heterogeneous uptake (Barrington and Meignan 2019). In the case of [^18^F]FDG PET/CT, various thresholds have been attempted to delineate tumours, although to date there is no consensus regarding optimal discriminating values (Barrington and Meignan 2019; Martín-Saladich et al. 2020). Recently, a new prognostic index was proposed to evaluate the outcome using MTV, age, and performance status (Mikhaeel et al. 2022). The combination of MTV using [^18^F]FLT has a potential incremental clinical value over [^18^F]FDG for detecting early relapse in these heterogeneous lymphoproliferative tumours. To the best of our knowledge, this approach has not been explored. For this purpose, disposal of reliable reference values for lesion delineation is a critical step to obtain quantitative information from PET/CT images.

Accordingly, we sought to propose and evaluate a segmentation threshold for [^18^F]FLT PET/CT images based on a reference tissue uptake on a cohort of patients with DLBCL that have been scanned at different stages of the treatment. It is out of the scope of this work to compare [^18^F]FDG versus [^18^F]FLT as probable prognostic value in early relapse of the disease.

## Methods

### Patients

This prospective study was designed and conducted at CEMIC University Hospital. It was approved by the institutional ethical committee and all patients signed an informed consent. We enrolled adult patients with DLBCL confirmed in II–IV stages without nervous system compromise and ECOG between 0 and 2. None of them received previous treatment.

### PET/CT protocol

The whole study was designed to use both [^18^F]FDG and [^18^F]FLT radiotracers. The time elapsed between each scan on a given patient does not exceeded 10 days. All patients were scanned using the same system (Gemini TF64; Philips Medical Systems, Eindhoven, The Netherlands) at the time of diagnosis (baseline PET), after 2 or 3 cycles of chemotherapy (interim PET or iPET), and at 2 weeks after the end of treatment (fPET) for each radiotracer at CEMIC University Hospital. The [^18^F]FLT administered activity was 1.8–2.6 MBq/kg body weight (Valda et al. 2022)﻿. All the acquisitions were performed 60–70 min after injection of each radiotracer and without use of contrast-enhanced CT.

### PET/CT data analysis

It was our goal to establish a threshold for the segmentation of malignant tissue uptake based on the uptake in a reference tissue, as done for [^18^F]FDG in the PERCIST criteria (Wahl et al. 2009). In order to investigate the feasibility of setting the liver or bone marrow as the reference tissue (Cysouw et al. 2017), we started by analysing their uptake stability throughout the treatment. Moreover, on both tissues, the analysis was performed by considering different definitions of the volume of interest (VOI), three for the liver and three for the bone marrow. Thus, for the liver, spherical VOIs of different diameters (29 mm, 41 mm, and 48 mm) were placed in the upper right lobe (segment VIII). For bone marrow, single and multiple vertebrae (T12, L3 and T10–T11–T12) were delineated on the CT image based on its Hounsfield units (HU) (Schreiber et al. 2014). From each of these six VOIs, mean SUV was extracted. For each kind of tissue, the different delineation methods were compared at each stage.

In order to quantify the hepatic uptake for each patient *j* at each stage (namely, baseline, iPET or fPET), the mean SUV (${\text{SUV}}_{H}^{{{\text{patient}} j, {\text{stage}}}}$) and its standard deviation (${\text{SD}}_{H}^{{{\text{patient}} j,{\text{stage}}}}$) in a spherical VOI of 29 mm diameter located in segment VIII of the liver were obtained. Analogously, to quantify bone marrow uptake for patient *j* at each stage, the mean SUV (${\text{SUV}}_{M}^{{{\text{patient}} j,{\text{stage}}}}$) and its standard deviation (${\text{SD}}_{M}^{{{\text{patient}} j,{\text{stage}}}}$) in T12 vertebra were obtained. For those patients who have been scanned at three stages, per cent relative change (${\text{RC}}$) of SUV with respect to the baseline stage was calculated for both tissues according to

1 $${\text{RC}}_{{{\text{tissue}}}}^{{{\text{patient}} j, {\text{stage}}}} = \frac{{{\text{SUV}}_{{{\text{tissue}}}}^{{{\text{patient}} j, {\text{stage}}}} - {\text{SUV}}_{{{\text{tissue}}}}^{{{\text{patient}} j,{\text{baseline}}}} }}{{{\text{SUV}}_{{{\text{tissue}}}}^{{{\text{patient}} j,{\text{baseline}}}} }} 100.$$

To establish whether liver or bone marrow could be the reference tissue for each patient, we computed the mean value of the uptake on each tissue (and its standard deviation) considering the data of all the patients and stages. We defined a normal uptake range of each tissue as its mean SUV plus/minus 1 standard deviation. If the hepatic uptake of patient *j* at stage *k* (${\text{SUV}}_{H}^{j,k}$) lies within the normal uptake range, then the liver can be taken to be the reference tissue. Likewise, if the bone marrow uptake of patient *j* at stage *k* (${\text{SUV}}_{M}^{j,k}$) lies within the normal uptake range, then the bone marrow can be taken to be the reference tissue.

The lesions, previously interpreted and reported by the two nuclear medicine physicians, were manually delineated by an experienced nuclear medicine technologist on the CT image using the LifeX software (Nioche et al. 2018). For each manually segmented lesion *i* in a patient *j*, the minimum SUV was recorded (${\text{SUV}}_{{{\text{min}}}}^{{{\text{patient}} j, i}}$); given that each VOI lesion is determined from the CT image, the variability of the minimum SUV associated with the VOI definition is highly moderated. The set of ${\text{SUV}}_{{{\text{min}}}}^{{{\text{patient}} j, i}}$, normalized to the uptake in a reference tissue, will be used to build a minimum global threshold from which two additional increasing quantities will be assessed as thresholding criteria. Therefore, the approach chosen in this work was to test different thresholds starting from a less restricted, but nevertheless well defined, value. For this purpose, let us consider a given stage and calculate the average ratio between ${\text{SUV}}_{\min }^{{{\text{patient}} j, i}}$ and both, the hepatic and the bone marrow uptakes. This average is performed on the total number of lesions segmented for the group of patients at the given stage, denoted by *N*_stage_. In this manner, a minimum threshold relative to hepatic uptake (${\text{RT}}_{H}^{{{\text{stage}}}}$) and to bone marrow uptake (${\text{RT}}_{M}^{{{\text{stage}}}}$) can be defined for each stage:

2 $${\text{RT}}_{H}^{{{\text{stage}}}} = \frac{1}{{N_{{{\text{stage}}}} }}\sum\limits_{i,j} {\frac{{{\text{SUV}}_{{{\text{min}}}}^{j,i} }}{{{\text{SUV}}_{H}^{{j,{\text{stage}}}} }}} ;\;{\text{RT}}_{M}^{{{\text{stage}}}} = \frac{1}{{N_{{{\text{stage}}}} }}\sum\limits_{i,j} {\frac{{{\text{SUV}}_{{{\text{min}}}}^{j,i} }}{{{\text{SUV}}_{M}^{{j,{\text{stage}}}} }} } .$$

Once a relative threshold (which is the same for all the patients) was established, the absolute individual threshold for each patient was computed. When employing the threshold based on a given reference tissue, we considered as pathologic every voxel whose SUV was higher than the corresponding minimum relative threshold (given in expression (2)) times the uptake of that reference tissue, specific for each patient. This is what we defined as the thresholding criterion 1. We also considered the possibility of classifying as pathologic every voxel whose SUV was higher than the corresponding relative threshold times the uptake of that reference tissue plus one standard deviation (criterion 2) or two standard deviations (criterion 3). For each patient *j*, these criteria for defining a threshold can be expressed, for each reference tissue and each stage, as:

- *Criterion 1* $${\text{RT}}_{{{\text{tissue}}}}^{{{\text{stage}}}} \times {\text{SUV}}_{{{\text{tissue}}}}^{{j,{\text{stage}}}}$$
- *Criterion 2* $${\text{RT}}_{{{\text{tissue}}}}^{{{\text{stage}}}} \times \left( {{\text{SUV}}_{{{\text{tissue}}}}^{{j,{\text{stage}}}} + 1 {\text{SD}}_{{{\text{tissue}}}}^{{j,{\text{stage}}}} } \right)$$
- *Criterion 3* $${\text{RT}}_{{{\text{tissue}}}}^{{{\text{stage}}}} \times \left( {{\text{SUV}}_{{{\text{tissue}}}}^{{j,{\text{stage}}}} + 2 {\text{SD}}_{{{\text{tissue}}}}^{{j,{\text{stage}}}} } \right)$$

We delineated all the lesions of all the patients at every stage using these criteria, being careful not to include any physiological uptake. In each case, the number of lesions was obtained. An experienced technician-physician team evaluated qualitatively the performance of each applied threshold according to its ability to correctly delineate the lesions previously reported on the CT images. This evaluation considered, for example, the loss of pathologic nodes and the merging of different lesions in the PET image. For a given patient, if both the hepatic and bone marrow uptake lay within the corresponding normal range, then the segmentation methods based on both tissues were tested.

Then, the criterion that showed the best performance for each stage was selected. In the case that criterion 2 or 3 had been selected, we reformulated its expressions in order to find a new relative threshold (designed as $\widetilde{RT}_{{{\text{tissue}}}}^{{{\text{stage}}}}$) that, applied to the mean value of the reference tissue (without adding any standard deviation), would result in the same absolute value for each patient. The purpose of this change was to obtain for criteria 2 and 3 expressions analogous to that of criterion 1. Namely, for each patient *j*, we looked for $\widetilde{{{\text{RT}}}}_{{{\text{tissue}}}}^{{j,{\text{stage}}}}$ such that

$${\text{RT}}_{{{\text{tissue}}}}^{{{\text{stage}}}} \times \left( {{\text{SUV}}_{{{\text{tissue}}}}^{{j,{\text{stage}}}} + n {\text{SD}}_{{{\text{tissue}}}}^{{j,{\text{stage}}}} } \right) = \widetilde{{{\text{RT}}}}_{{{\text{tissue}}}}^{{j,{\text{stage}}}} \times {\text{SUV}}_{{{\text{tissue}}}}^{{j,{\text{stage}}}}$$

where *n* indicates the number of added standard deviations (1 or 2). Then, $\widetilde{{{\text{RT}}}}_{{{\text{tissue}}}}^{{{\text{stage}}}}$ is obtained by averaging over all the patients:

3 $$\widetilde{{{\text{RT}}}}_{{{\text{tissue}}}}^{{{\text{stage}}}} = \frac{{{\text{RT}}_{{{\text{tissue}}}}^{{{\text{stage}}}} }}{{N_{{{\text{pat}}_{{{\text{stage}}}} }} }}\sum\limits_{j} {\frac{{\left( {{\text{SUV}}_{{{\text{tissue}}}}^{{j,{\text{stage}}}} + n {\text{SD}}_{{{\text{tissue}}}}^{{j,{\text{stage}}}} } \right)}}{{{\text{SUV}}_{{{\text{tissue}}}}^{{j,{\text{stage}}}} }}}$$

where $N_{{{\text{pat}}_{{{\text{stage}}}} }}$ is the number of patients in the considered stage. It can be seen that this expression is consistent with that of criterion 1 if we put *n* = 0, i.e. ${\text{RT}}_{{{\text{tissue}}}}^{{{\text{stage}}}} = \widetilde{{{\text{RT}}}}_{{{\text{tissue}}}}^{{{\text{stage}}}}$.

## Results

### Patients

We included 23 patients (11 males and 12 females) with confirmed DLBCL who underwent PET/CT studies between February 2018 and October 2019. The total number of planned PET/CT acquisitions using [^18^F]FLT was 69. This ideal design was not achieved due to logistic difficulties associated with the radiopharmaceutical and general clinical condition of the patients. However, all the patients had at least two PET studies in order to meet the objectives of this study.

From the 23 patients scanned with [^18^F]FLT at different stages 18 of them were scanned before starting the treatment (baseline PET), 17 patients had a PET scan after receiving 2 or 3 cycles of treatment (iPET) and 12 patients had an end-of-treatment PET scan (fPET). The full acquisition scheme was completed in 8 patients. Table 1 shows the study distribution per patient.

**Table 1:** Distribution of studies per patient, including [^18^F]FDG and [^18^F]FLT scans, at baseline, interim and end-of-treatment stages

Patient ID	Baseline		Interim		End-of-treatment	
	FDG	FLT	FDG	FLT	FDG	FLT
1	×	−	×	×	×	×
2	×	×	−	−	−	−
3	×	×	−	×	−	−
4	×	×	×*	×*	×	×
5	×	−	−	×	×	×
6	×	×	×	−	×	−
7	×	×*	×*	−	−	×*
8	×	×	×	×	−	×
9	×	×	×	×	−	−
10	×	×	−	×	×*	×
11	×	×	−	×	×*	×*
12	×	×	×	×	×	×
13	−	−	×*	×*	×	−
14	×	×	×*	×*	×*	×*
15	×	×	×	×	×	×
16	−	×	×	×	−	−
17	×	×*	−	−	−	−
18	×	−	×	×	−	−
19	×	−	−	×*	×*	× *
20	×	×	−	−	−	−
21	×	×	−	−	−	−
22	×	×	−	×*	×	×
23	×	×	×	×	−	−
Number of scans:	21	18	13	17	12	12

×: Scan performed and segmented

×*: Scan performed without abnormal uptake (no lesions to segment)

−: Scan not performed

### Reference tissue stability

**Fig. 1:**
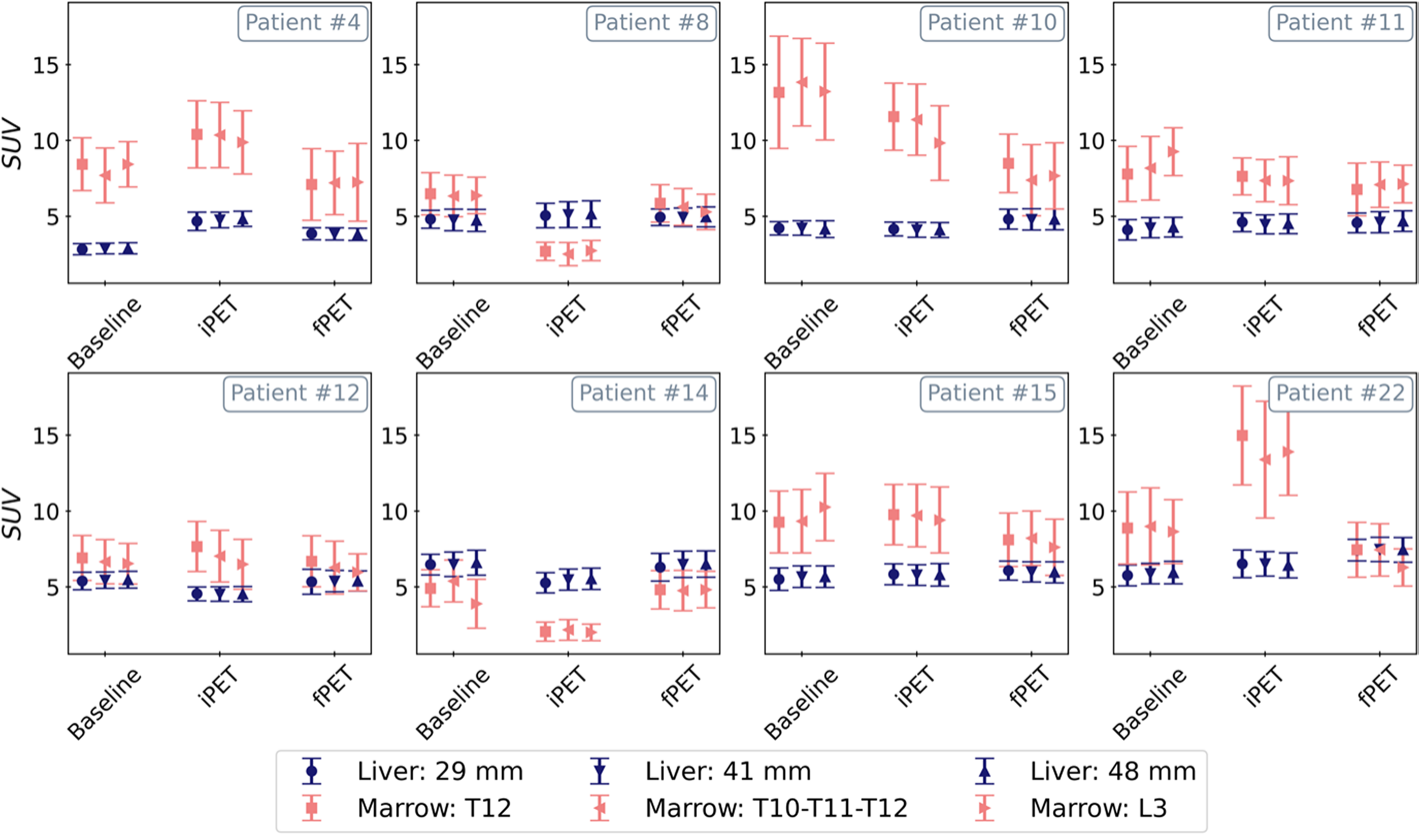
Comparison of different VOIs used to quantify the reference tissues uptake: spherical VOIs of 29 mm, 41 mm and 48 mm diameter placed in segment VIII of the liver (to quantify hepatic uptake) and vertebrae T12, L3 and the set T10–T11–T12 (to quantify bone marrow uptake). Each point: mean SUV ± 1 standard deviation. Each plot represents one patient that has been scanned at every stage

Figure 1 depicts the results of the 6 different methods employed to quantify the reference uptake on a patient-level basis among the 8 patients who completed three PET scans (baseline, iPET and fPET). At each stage, the mean SUV of each VOI is plotted: spherical VOIs of 29 mm, 41 mm and 48 mm diameters placed in segment VIII of the liver (to quantify hepatic uptake) and vertebrae T12, L3 and the set T10–T11–T12 (to quantify bone marrow uptake). As shown, the three VOIs placed in the liver are equivalent at every stage intra-patient for all the patients, and therefore, any of them could be used. Mostly, this pattern is repeated for the bone marrow using the three VOIs. Hence, we defined the hepatic uptake as the mean SUV in a spherical volume of 29 mm diameter and the bone marrow uptake as the mean SUV in the T12 vertebra.

**Fig. 2:**
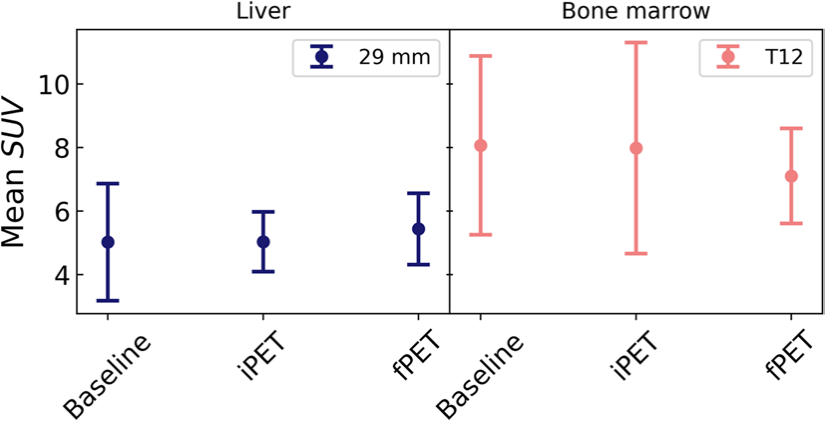
Mean hepatic (spherical VOI 29 mm in diameter) and bone marrow (T12) uptake for all patients at each stage

**Fig. 3:**
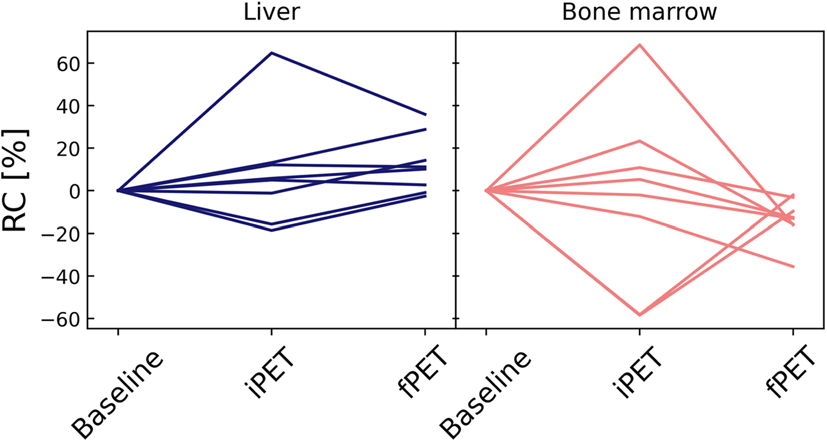
Relative changes with respect to baseline PET at each stage for the patients who have been scanned three times (each line represents a patient)

Mean and standard deviation for the hepatic uptake (determined on the spherical VOI 29 mm in diameter) and for the bone marrow uptake (determined on T12) were obtained for all available patients at each stage. Differences between means of these multiple groups were evaluated using one-way analysis of variance (ANOVA). Results are shown in Table 2 and Fig. 2. As can be seen, both the hepatic and bone marrow uptake are extremely stable, on average, along the treatment, allowing us to use any of them as reference tissue. Figure 3 shows the individual relative changes with respect to the baseline PET at each stage for those patients having the three scans.

**Table 2:** Mean uptake (± 1 sd) values in liver and bone marrow and ANOVA results

	Baseline	iPET	fPET	*p*-value
Liver	5.0 ± 1.8	5.0 ± 0.9	5.4 ± 1.1	0.68
Bone marrow (T12)	8.1 ± 2.8	8.0 ± 3.3	7.1 ± 1.5	0.61

### Normal uptake in liver and bone marrow

**Fig. 4:**
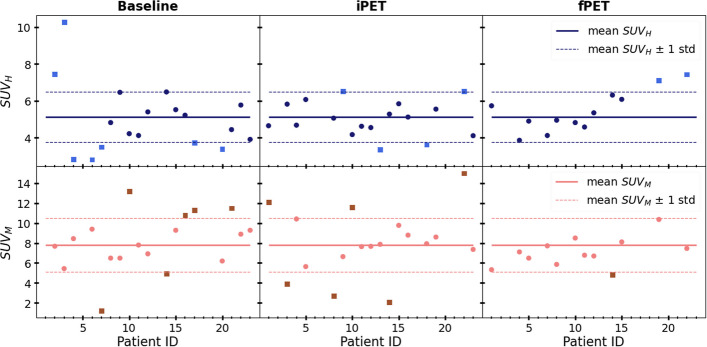
Hepatic (upper) and bone marrow (lower) uptake for each patient at baseline, iPET and fPET. The solid line indicates the mean hepatic uptake (upper) and the mean bone marrow uptake (lower) of all patients and stages. The dashed lines indicate the normal uptake range, defined as the mean values plus/minus one standard deviation. The squares indicate those patients that are not within the normal uptake range

Figure 4 shows the hepatic (upper row) and bone marrow (lower row) uptake for each patient at each stage. It is also shown the range of normal uptake, defined as the mean uptake of all patients and stages plus/minus one standard deviation. Thus, for the liver, the normal uptake range is 5.1 ± 1.4, and for the bone marrow, 7.8 ± 2.7. According to this definition, those SUV values plotted as circles lie within the normal range, whereas the squares indicate those patients whose uptake lie outside the normal range.

### Minimum relative threshold

**Fig. 5:**
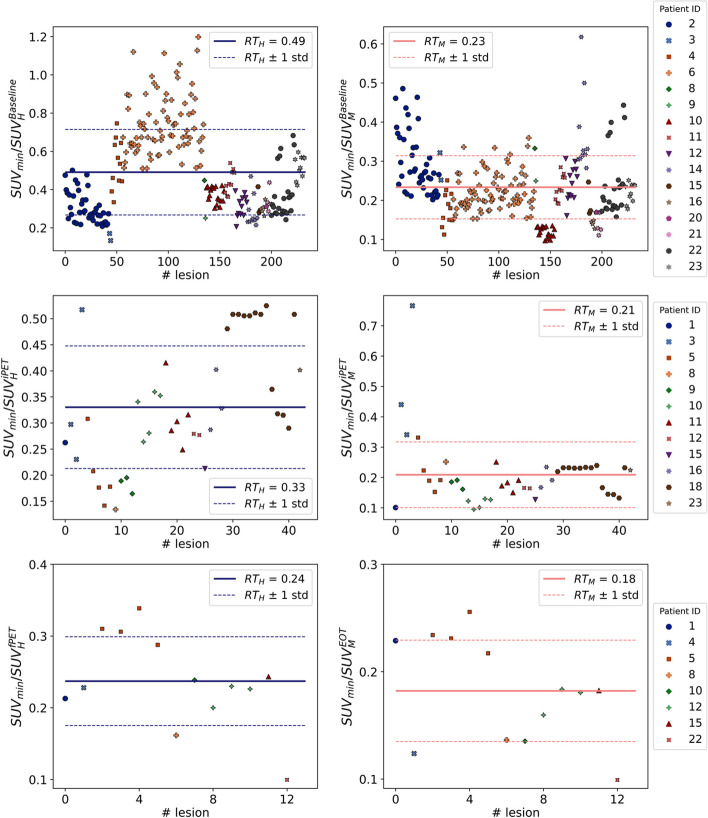
Ratios between the minimum SUV of every manually delineated lesion and the hepatic (left) and bone marrow (right) uptakes of the corresponding patient, for each stage: baseline (top row), iPET (intermedial row) and fPET (bottom row). The solid line indicates the relative threshold obtained by averaging those values, and the dashed lines delimit the region within one standard deviation

For each stage, a minimum threshold relative to hepatic uptake (${\text{RT}}_{H}^{{{\text{stage}}}}$) and to bone marrow uptake (${\text{RT}}_{M}^{{{\text{stage}}}}$) was obtained as the average value of the ratios between ${\text{SUV}}_{\min }^{j,i}$ of every manually segmented lesion *i* and the hepatic and bone marrow uptakes (${\text{SUV}}_{H}^{{j,{\text{stage}}}}$ and ${\text{SUV}}_{M}^{{j,{\text{stage}}}}$, respectively) of the corresponding patient *j* following the expressions given in (2). The total number of lesions (*N*_stag*e*_) found at each stage were: *N*_baseline_ = 235, *N*_iPET_ = 43 and *N*_fPET_ = 13. The ratios ${\text{SUV}}_{\min }^{j,i} {\text{/SUV}}_{{{\text{tissue}}}}^{{j,{\text{stage}}}}$ are plotted in Fig. 5, and the derived minimum relative thresholds are shown in Table 3.

**Table 3:** Minimum thresholds relative to liver and bone marrow found at each stage

Minimum relative threshold		
Stage	Liver (%)	Bone marrow (%)
Baseline	49	23
iPET	33	21
fPET	24	18

### Assessment of the three segmentation criteria

We analysed and compared three criteria for determining the minimum SUV of a voxel to be considered as belonging to a pathologic tissue. To that end, we segmented all the lesions employing each of the proposed thresholds and analysed the resulting boundaries for the lesions. The performance of each method was evaluated qualitatively according to its ability to correctly delineate the lesions by comparing the thresholding results with that obtained by an experienced technician-physician team. Table 4 shows the percentage of cases for which one criterion was qualitatively preferred over the others by the experienced team at each stage. Besides, we compared the number of detected lesions (with at least one voxel). Table 5 shows the number of detected lesions employing each method at each stage. As can be seen from Tables 4 and 5, all the methods are capable of finding more than 86% of the lesions. However, there is one procedure that is clearly preferred over the others at each stage.

**Table 4:** Comparison of the three different criteria for delineating the lesions: qualitative performance

Stage	Reference tissue†	Qualitative performance*		
		Criterion 1 (%)	Criterion 2 (%)	Criterion 3 (%)
Baseline	Liver	9.0	0.0	91.0
	Bone marrow	8.3	8.3	83.3
iPET	Liver	91.0	0.0	9.0
	Bone marrow	66.7	0.0	33.3
fPET	Liver	0.0	71.0	29.0
	Bone marrow	33.3	44.4	22.2

*Qualitative performance expressed in terms of the percentage of cases for which one method was preferred over the others

^†^For each reference tissue, we considered only those patients whose uptake lay within the normal range

**Table 5:** Comparison of the three different criteria for delineating the lesions: number of detected lesions

Stage	Reference tissue^†^	Number of segmented lesions*			
		Reported	Criterion 1	Criterion 2	Criterion 3
Baseline	Liver	111	109 (98%)	106 (95%)	104 (94%)
	Bone Marrow	211	209 (99%)	204 (97%)	195 (92%)
iPET	Liver	30	30 (100%)	27 (90%)	27 (90%)
	Bone Marrow	36	36 (100%)	36 (100%)	33 (92%)
fPET	Liver	12	12 (100%)	12 (100%)	12 (100%)
	Bone Marrow	14	14 (100%)	14 (100%)	12 (86%)

*Number of detected lesions following the different thresholding criteria. Percentage with respect to the number of reported lesions is given in parentheses

^†^For each reference tissue, we considered only those patients whose uptake lay within the normal range

**Fig. 6:**
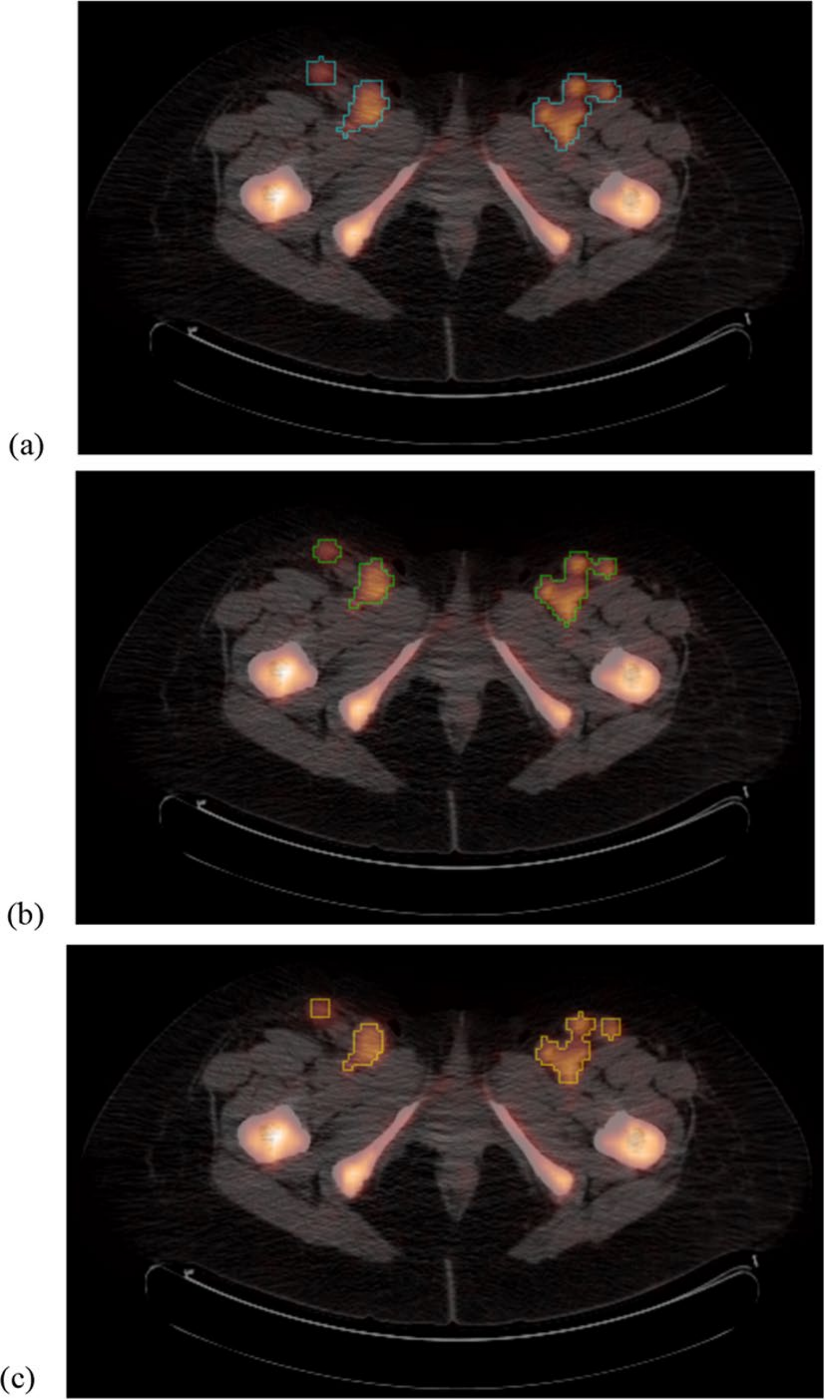
Example for comparing the limits of a lesion found by the three different criteria in a baseline PET/CT: **a** criterion 1; **b** criterion 2; and **c** criterion 3

As an example, Fig. 6 shows the results obtained by applying each of the three thresholding criteria on a set of lesions in patient P1 on its baseline PET. Taking all these results into account, we concluded that the best method for determining the minimum SUV of a voxel to be considered as malignant is different at each stage: criterion 3 at baseline, criterion 1 for iPET and criterion 2 for fPET, regardless of the reference tissue. In this way, and applying the expression given in 3, we obtained the final thresholds presented in Table 6. When employing the final threshold based on a given reference organ, a voxel should be considered as pathologic if its SUV is higher than the corresponding final relative threshold times the uptake of that reference organ.

**Table 6:** Final thresholds relative to liver and bone marrow found at each stage

Stage	Final relative threshold	
	Liver (%)	Bone marrow (%)
Baseline	62	35
iPET	33	21
fPET	27	22

## Discussion

The aim of the present study was to search for threshold values of malignancy in [^18^F]FLT studies based on the uptake of a reference tissue. In particular, in accordance with previous results (Cysouw et al. 2017), we investigated the feasibility of choosing liver and bone marrow as reference tissues and evaluated different types of VOIs to quantify their uptakes. We found the reference tissues uptake to have low dependency on the different sizes and locations of the VOIs, allowing us to use either of them. Regarding the liver reference, it was decided to continue defining the hepatic uptake as the mean SUV in a spherical volume of 29 mm diameter placed in the upper right lobe of the liver since it is the closest to that proposed by PERCIST criteria (Wahl et al. 2009). Relating to the use of the bone marrow as reference tissue, it was observed low variability uptake among the single vertebra (T12 or L3) and the group T10–T11–T12 through the time for each stage. This outcome drove us to decide the use of the T12 as the bone marrow reference, defined as its mean SUV uptake. Should the case any of them were compromised because of the illness, there is the possibility to use one of the other two options.

After averaging over all patients, both the hepatic and bone marrow uptakes were stable across the different treatment stages, suggesting the adequacy for using these tissues as reference independent of the phase or surveillance timepoint.

Given that the quantification of liver uptake is much simpler than that of bone marrow, we propose to use this tissue as reference every time its uptake lies within normal ranges. Whenever the hepatic uptake of a given patient is not within the expected range, but the bone marrow uptake is, the bone marrow should be taken to be the reference tissue. We have found in our study three patients for which both the hepatic and the bone marrow uptake lay outside the corresponding normal range. This occurs at baseline with two patients (ID 7 and 17). However, they show no lesions in their scans and therefore, they were not included in the relative threshold computation nor in the segmentation method evaluation. The same happens with a different patient (ID 21) at iPET.

We found that it is not possible to use the same relative threshold throughout the treatment. Instead, the fraction between the minimum SUV of a voxel to be considered as malignant and the reference tissue uptake decreases along the treatment. The causes leading to this decrease cannot be completely elucidated within the frame of this study. Bias in the small patient group that completed the full acquisition scheme (8 patients) and correlations to response to the treatment cannot be excluded. However, we observed that as treatment progresses, the number of lesions reduces (as can be seen in Fig. 5) as well as their size, the small lesions being prevalent at fPET. As a result, this fact makes the uptake quantification more affected by the partial volume effect underestimating the SUV. The variety of lesions shapes make it difficult for simple contrast recovery methods to be applied. In this study, we do not apply any contrast recovery techniques.

It is important to mention an important asset of this work. Many of the patients that participated in this study have been scanned twice or even three times along the treatment, and we therefore have complete information to compare the different stages.

We are aware of the limitations of this preliminary study that attempted to find reference values of [^18^F]FLT for applying in the clinical practice. On the one hand, this study was performed in one centre and therefore, our results may change when employing different scanners. Besides, as the study involved a small sample size, the proposed method was defined using all the available data and tested on the same scan set. Therefore, it would be necessary to extend this study by including new patients in order to test the method on new data and minimise potential bias.

Despite this small sample size, we believe that the methodology presented in this work could help in establishing robust thresholds on [^18^F]FLT PET/CT images in DLBCL. Defining a threshold criterion that enables the lesions segmentation will make it possible to accurately compute the MTV and evaluate its prognostic power. These results could also serve as part of artificial intelligence methods (Visvikis et al. 2022), such as those already being proposed for [^18^F]FDG studies in DLBCL (Ferrández et al. 2023; Kuker et al. 2022; Capobianco et al. 2021).

## Conclusion

Based on the methodology applied to [^18^F]FLT PET/CT images during staging and follow-up of patients with DLBCL, we obtained threshold values for the segmentation of lesions. The thresholds, normalized to liver and bone marrow uptake, were obtained for each stage and could be used in clinical practice oncology.

## Data Availability

The datasets used and/or analysed during the current study are available from the corresponding author on reasonable request.

## Abbreviations

[^18^F]FDG: [^18^F]fluorodeoxyglucose
[^18^F]FLT: [^18^F]fluorothymidine
CT: Computed tomography
DLBCL: Diffuse large B-cell lymphoma
ECOG: Eastern Cooperative Oncology Group (with reference to patient performance status)
fPET: End-of-treatment PET
HU: Hounsfield units
ID: Patient identification number (within formulae it is equivalent to index *j*)
iPET: Interim PET
MTV: Metabolic tumour volume
PERCIST: Positron emission tomography response criteria in solid tumours
VOI: Volume of interest
PET: Positron emission tomography
${\text{RC}}_{{{\text{tissue}}}}^{{{\text{patient}} j, {\text{stage}}}}$: Per cent relative change of the mean SUV with respect to the baseline stage measured on a given tissue (liver or bone marrow), for a given patient *j* at a given stage (baseline, iPET or fPET)
${\text{SD}}_{H}^{{{\text{patient}} j,{\text{stage}}}}$: Standard deviation of ${\text{SUV}}_{H}^{{{\text{patient}} j,{\text{ stage}}}}$
${\text{SD}}_{M}^{{{\text{patient}} j,{\text{stage}}}}$: Standard deviation of ${\text{SUV}}_{M}^{{{\text{patient}} j,{\text{stage}}}}$
SUV: Standardized uptake value
${\text{SUV}}_{H}^{{{\text{patient}} j, {\text{stage}}}}$: Mean SUV on a VOI placed on the liver, for a given patient *j* on an image acquired at a given stage (baseline, iPET or fPET)
${\text{SUV}}_{M}^{{{\text{patient}} j,{\text{stage}}}}$: Mean SUV on a VOI placed on the bone marrow, for a given patient *j* on an image acquired at a given stage (baseline, iPET or fPET)
${\text{SUV}}_{\min }^{{{\text{patient}} j, i}}$: Minimum SUV measured on lesion *i* belonging to patient *j*
${\text{RT}}_{{{\text{tissue}}}}^{{{\text{stage}}}}$: Relative threshold with respect to the reference tissue uptake (where tissue may be *H* for liver or *M* for bone marrow) at a given stage (baseline, iPET or fPET)
$\widetilde{{{\text{RT}}}}_{{{\text{tissue}}}}^{{{\text{stage}}}}$: Relative threshold with respect to the reference tissue uptake at a given stage that considers (in an averaged way) the addition of 0, 1 or 2 standard deviations ${\text{SD}}_{{{\text{tissue}}}}^{{j,{\text{stage}}}}$ to ${\text{SUV}}_{{{\text{tissue}}}}^{{j,{\text{stage}}}}$ (tissue: *H* for liver or *M* for bone marrow)

## References

[CR1] Barrington SF, Meignan M (2019). Time to prepare for risk adaptation in lymphoma by standardizing measurement of metabolic tumor burden. J Nucl Med.

[CR2] Buck AK, Bommer M, Stilgenbauer S (2006). Molecular imaging of proliferation in malignant lymphoma. Can Res.

[CR3] Capobianco N, Meignan M, Cottereau A-S (2021). Deep-learning ^18^F-FDG uptake classification enables total metabolic tumor volume estimation in diffuse large B-cell lymphoma. J Nucl Med.

[CR4] Cysouw M, Kramer GM, Frings V (2017). Baseline and longitudinal variability of normal tissue uptake values of [18F]-fluorothymidine-PET images. Nucl Med Biol.

[CR5] Ferrández MC, Golla SSV, Eertink JJ (2023). An artificial intelligence method using FDG PET to predict treatment outcome in diffuse large B cell lymphoma patients. Sci Rep.

[CR6] Kuker RA, Lehmkuhl D, Kwon D (2022). A deep learning-aided automated method for calculating metabolic tumor volume in diffuse large B-cell lymphoma. Cancers.

[CR7] Martín-Saladich Q, Reynés-Llompart G, Sabaté-Llobera A (2020). Comparison of different automatic methods for the delineation of the total metabolic tumor volume in I–II stage Hodgkin lymphoma. Sci Rep.

[CR8] McKinley ET, Ayers GD, Smith RA (2013). Limits of [18F]-FLT PET as a biomarker of proliferation in oncology. PLoS ONE.

[CR9] Mengüç MU, Mehtap Ö, Görür GD (2021). The role of interim PET/CT on survival in diffuse large B cell lymphoma. Clin Lymph Myeloma Leuk.

[CR10] Mikhaeel NG, Heymans MW, Eertink JJ (2022). Proposed new dynamic prognostic index for diffuse large B-cell lymphoma: international metabolic prognostic index. J Clin Oncol.

[CR11] Minamimoto R, Fayad L, Advani R (2016). Diffuse large B-cell lymphoma: prospective multicenter comparison of early interim FLT PET/CT versus FDG PET/CT with IHP, EORTC, deauville, and PERCIST criteria for early therapeutic monitoring. Radiology.

[CR12] Nioche C, Orlhac F, Boughdad S (2018). LIFEx: a freeware for radiomic feature calculation in multimodality imaging to accelerate advances in the characterization of tumor heterogeneity. Cancer Res.

[CR13] Sasanelli M, Meignan M, Haioun C (2014). Pretherapy metabolic tumour volume is an independent predictor of outcome in patients with diffuse large B-cell lymphoma. Eur J Nucl Med Mol Imag.

[CR14] Schreiber JJ, Anderson PA, Hsu WK (2014). Use of computed tomography for assessing bone mineral density. Neurosurg Focus.

[CR15] Song MK, Chung JS, Shin HJ (2012). Clinical significance of metabolic tumor volume by PET/CT in stages II and III of diffuse large B cell lymphoma without extranodal site involvement. Ann Hematol.

[CR16] Spaepen K, Stroobants S, Dupont P (2003). [18F]FDG PET monitoring of tumour response to chemotherapy: does [18F]FDG uptake correlate with the viable tumour cell fraction?. Eur J Nucl Med Mol Imag.

[CR17] Valda A, Bastianello M, Casale G (2022). [18F]Fluorothymidine preclinical study in non-human primate. Dosimetry and Biodistribution. Medicina (Buenos Aires).

[CR18] Visvikis D, Lambin P, Beuschau Mauridsen K (2022). Application of artificial intelligence in nuclear medicine and molecular imaging: a review of current status and future perspectives for clinical translation. Eur J Nucl Med Mol Imag.

[CR19] Wahl RL, Jacene H, Kasamon Y, Lodge MA (2009). From RECIST to PERCIST: evolving considerations for PET response criteria in solid tumors. J Nucl Med.

